# 521 Maintaining Central Line Patency in Burned Children

**DOI:** 10.1093/jbcr/irac012.152

**Published:** 2022-03-23

**Authors:** Olivia Arredondo, Tina L Palmieri, Soman Sen, David G Greenhalgh, Kathleen S Romanowski

**Affiliations:** Shriners Hospital for Children Northern California, Sacramento, California; UC Davis and Shriners Hospitals for Children Northern California, Sacramento, California; UC Davis, Sacramento, California; University of California - Davis, Sacramento, California; University of California, Davis and Shriners Hospitals for California Northern California, Sacramento, California

## Abstract

**Introduction:**

Children with major burn injury frequently require prolonged central venous access to assure appropriate fluid management and pain control. Central venous catheters in children frequently develop clots that prevent drug administration, requiring administration of tissue plasminogen activator (TPA). The purpose of this study was to identify the frequency and efficacy of TPA use in burned children with central venous catheters (CVC).

**Methods:**

This retrospective chart review evaluated all children requiring CVC admitted to our tertiary pediatric burn center from 2018-2019. Data collected included patient demographics (age, burn size, hospital length of stay (LOS)), catheter-related data (number of central lines, lines replaced due to clotting), TPA administration (number of times administered, successful TPA administrations, how often repeated), and line clotting data (time from insertion to clot, interval between TPA order and administration).

**Results:**

In 2018, 116 lines were place in 49 children with mean age of 8.4 years and mean burn size of 29%, intensive care unit LOS was 24 days. TPA was infused in 20% of lines to relieve obstruction and was successful in relieving the clot in 21% (5/23). The interval between identification of the obstructed line to TPA order was 191 minutes, with the administration of TPA 83 minutes after order placement. The average time from identification of obstruction to TPA administration was 257 minutes. In 2019, 150 lines were place in 65 children with mean age of 5.2 years and mean burn size of 25%, LOS was 13 days in the PICU. TPA was infused in 5% of lines to relieve obstruction and was successful in relieving the clot in 0 % (0/8). The interval between identification of the obstructed line to TPA order was 117 minutes, with the administration of TPA 49 minutes after order placement. The average time from identification of obstruction to TPA administration was 158 minutes.

**Conclusions:**

The incidence of obstruction in pediatric central venous catheters in our unit decreased from 26% in 2018 to 3% in 2019. TPA was successful in clot resolution in only 5% (2018), and 0% (2019) . Based on our results, we targeted areas for improvement including: Standing order for TPA; staff education on TPA use; decreasing our average time to identify, order, and administer TPA; and standardizing the frequency of flushing unused central venous catheter lumens with heparinized saline flush.

